# Synergistic and Antagonistic Effects of Piperonyl Butoxide in Fipronil-Susceptible and Resistant Rice Stem Borrers, *Chilo suppressalis*

**DOI:** 10.1673/031.010.14142

**Published:** 2010-10-21

**Authors:** Qingchun Huang, Yunfei Deng, Taisong Zhan, Yuan He

**Affiliations:** Shanghai Key laboratory of Chemical Biology, School of Pharmacy, East China University of Science and Technology, Shanghai, China

**Keywords:** synergism, resistance, phenylpyrazole insecticide

## Abstract

Using the phenylpyrazole insecticide, fipronil for selection in the laboratory, a resistant Wenzhou strain of the rice stem borer, *Chilo suppressalis* (Walker) (Lepidoptera: Crambidae) had an LD_50_ at least 45.3 times greater than the susceptible Anhui strain. The realized resistant heritability (*h*^2^) of 0.213 showed that the tolerant phenotype was moderately heritable and had potential to develop higher tolerance to fipronil. Piperonyl butoxide decreased the effects of fipronil on the mortality of the susceptible larvae with 0.27–0.44 times synergistic rates, but increased the toxicity of fipronil on the resistant larvae with 1.85–2.53 times synergistic rates as compared to that of fipronil alone. The inhibitory effect of piperonyl butoxide on the activity of microsomal *O*-demethylase was greater in susceptible larvae than in the resistant larvae. The differential synergism of fipronil by piperonyl butoxide in the susceptible and resistant *C. suppressalis* may be caused by the reduced penetration of fipronil in the lab-selected Wenzhou strain.

## Introduction

The rice stem borer, *Chilo suppressalis* Walker (Lepidoptera: Crambidae), one of the economically most important rice pest insects in China, developed high levels of resistance to organophosphate and nereistoxin insecticides because of their long use in the field (Li et al. 2001; Han et al. 2002; Qu et al. 2003). At the end of 1990s, the phenylpyrazole insecticide, fipronil [(±)-5-amino-1-(2,6-dichloro-α,α,α-trifluoro *-p*-toly)-4-trifluoromethyl-sulfinylpyra- zole-3-carbonitrile], was used as an alternative agent to control the borer and showed excellent effects. Recently, there have been a few reports of *C. suppressalis* with low or middle level of resistance to fipronil in the field (Cao et al. 2004; Jing et al. 2005). In fact, high levels of fipronil resistance have been monitored in many other insects such as *Plutella xylostella, Musca domestica, Oulema oryzae* and *Drosophila simulans* (Sayyed et al. 2004; Kristensen et al. 2004; Ueno et al. 2005). The mutation of *Rdl* genes was responsible for conferring high level of fipronil resistance in *D. simulans* (Goff et al. 2005). Hence, the potential exists for high level of firponil resistance to develop in *C. suppressalis*.

Fipronil has a novel mode of action that disrupts chloride ion flow by interacting with the *γ*-aminobutyric acid gated chloride ionophore of the central nervous system (Ikeda et al. 2003; Zhao et al. 2005). Insects are able to utilize cytochrome P450-mediated oxidative metabolism to degrade fipronil to the oxidated fipronil-sulfone, a predominant metabolite with higher toxicity than fipronil itself (Durham et al. 2001; Hainzl et al. 1998; Scharf et al. 2000; Zhao et al. 2005). A synergistic test is a strategy employed to determine whether a particular metabolic enzyme is involved as an insecticidal resistance mechanism (Miranda et al. 1998). Piperonyl butoxide (PBO) acts as a synergist of insecticides by inhibiting the cytochrome P450-mediated metabolism of the insecticide (Jones 1998) so fipronil has the potential to be antagonized by piperonyl butoxide. In this paper, the resistant strain of *C. suppressalis* was selected with fipronil under laboratory conditions, and the resistant risk was assessed by the heritability parameter. The properties of microsomal *O*-demethylase, extracted from susceptible and resistant larvae were determined and the synergistic effects of piperonyl butoxide were also conducted. The aims were to obtain some insight into the physiological changes of the fipronil-resistant borer to guide the appropriate application of fipronil and delay the development of fipronil resistance in *C. suppressalis*.

## Materials and methods

### Insects

The Anhui strain of *C. suppressalis* was originally collected from a mountain area of Taihu county, Anhui province in 2000, where little pesticide has been traditionally used for rice pest control. The strain was maintained in the laboratory without exposure to any insecticide. The Wenzhou strain was collected from Wenzhou county, Zhejiang province in 2005, where fipronil had been the main insecticide used to control *C. suppressalis* in recent years. The strains were reared on rice seedling using the protocol of Shang et al (1979). The rearing conditions were 28 ± 1° C, 70–80% RH and a photoperiod of 16:8 L:D. Fourth instar larvae were used for experiments.

### Chemicals

Fipronil (87% purity) was obtained from Bayer Crop Science (www.bayercropscience.com). PBO was purchased from Fluka (www.sigmaaldrich.com). Both were dissolved in acetone as stock solution for experimental use. PMSF was purchased from E. Merck, (www.syngentacropprotection.com). NADPH and PTU were from Sigma Chemical (www.sigmaaldrich.com). EDTA, DTT and *p*-NA were from the Shanghai Reagent Factory (www.reagent.com.cn).

### Selection experiment

The susceptible strain of *C. suppressalis* to fipronil was obtained from the Anhui strain by using single-pair mating of adults. Single egg masses were collected and placed in different containers. After the larvae hatched, some individuals from each mass were randomly selected and treated topically with a dose of fipronil corresponding to the LD_20_ that was obtained from the experiments with their parental generation. If the individuals died following this treatment, others from the same egg mass were used as the susceptible strain, and the LD_20_ value was determined for them.

For establishment of the resistant strain, the adults from the Wenzhou strain were allowed to mate and oviposit, and egg masses were collected. Larvae were treated with doses of fipronil corresponding to the experimentally derived LD_70_ of their parental generation. Survivors were selected as the resistant strain, and the LD_70_ value was determined for them. The tolerance of the selected population to fipronil was assessed by bioassay and the heritability parameters were calculated using the formulae presented by Falconer (1989) and Tabashnik (1992): *h*^2^ = R/S, where *h^2^* is the realized heritability. R is the response to selection [R = (initial LD_50_ - final LD_50_)^-n^], the difference between the mean phenotype of the selected offspring and the parental generation before selection. S is the selection differential (S = *i* × *δ*_p_), the difference between the mean phenotype of the selected parents and the parental generation before selection. *i* is intensity of selection [*i* = 1.583 - 0.0193336*P* + 0.0000428*P*^2^ + 3.65194/*P* (10 < *P* < 80)]. *P* is the mean survival percentage. *δ*_p_ is the phenotypic standard deviation (*δ*_p_ = [1/2(initial slope + final slope)]^-1^).

### Bioassay protocol

Stock solutions of fipronil and PBO were diluted with acetone to the required concentrations, and then a droplet of 1 µl of the diluted solutions was applied topically on the middle-thoracic notum of the fourth-instar larvae (9–11mg) by using a hand microapplicator (Burkard, www.burkard.co.uk). Bioassays were repeated three times with 15 larvae per replicate for each concentration. Larvae treated with acetone only served as controls. The results were monitored at 48 h posttreatment. Dead larvae were counted if no response was obtained after being probed with a pin. Larvae mortalities were corrected using Abbott's formula (Abbott 1925). The data were subjected to probit analysis as the means with their standard errors and the LD_50_ values were determined (Finney 1971).

In the synergism experiments, the borers were topically treated by a droplet of 1 µl of 3.0, 6.0 and 15.0 g litre^-1^ PBO for 1 h, 3 h, 6 h, 9 h and 18 h prior to fipronil application, respectively. The treated larvae were then reared and the mortality was recorded at 48 h. The synergism ratio (SR) was expressed as the LD_50_ value of fipronil alone that was divided by the LD_50_ value of fipronil plus PBO.

### Microsomal *O*-demethylase assays

Microsomal *O*-demethylase (MOD) activity was conducted by the method described by Yang et al. (2004). Ten fourth-instar larvae were homogenized in 1 ml of ice-chilled phosphate buffer (0.1 M, pH 7.8) supplemented with 1.0 m*M* EDTA, 1.0 mM DTT, 1.0 mM PTU and 1.0 mM PMSF, the homogenate was centrifuged at 12,000 g for 15 min at 0° C, and the supernatant was recentrifuged at 100,000 g for 1 h at 0° C, then the pellet of microsomes was resuspended in the homogenization buffer as the enzyme extract. The reaction mixture contained 0.4 ml of phosphate buffer (0.1 M, pH 7.8), 0.25 ml of 6.0 mM *p*-NA and 90 µl enzyme extract. After preincubation for 5 min at 34° C, 0.25 ml of 2.0 mM NADPH was added to start the reaction, and the mixture was shaken during incubation at 34°C for 30 min. All tests were replicated three times. The change rate of optical density per minute at 405 nm was recorded using a Beckman spectrophotometer (www.beckmancoulter.com). The activity was measured as mOD · min^-1^. Protein content was determined by the Bradford (1976) method using bovine serum albumin as the standard.

### Data analysis

PoloPlus software (**LeOra Software**, www.leorasoftware.com) was used for probit analysis of the dose response data and calculations of LD_50_ values. Other data were subjected to analysis of variance (ANOVA) and means separated using the least significant difference (LSD) test. The level of significance (*P*) was set at 5%.

## Results

### Realized heritability (*h* ^2^) of resistance in *C. suppressalis*

After six generations of selection in response to fipronil the LD_50_ of the resistant Wenzhou strain changed from 15.6 to 172.1 ng/larva. The LD_50_ of fipronil for the susceptible Anhui strain gradually decreased from 7.3 to 3.8 ng/larva and then was stable. The resistant Wenzhou strain developed 45.3-fold resistance to fipronil. As shown in Table 1, the realized heritability (*h*^2^) of resistance to fipronil in the Wenzhou strain was 0.213 by probit analysis, indicating that the tolerant phenotypes in the population were moderately heritable and the population had potential to increase to higher levels of resistance to fipronil under laboratory selection.

### Dose-effect synergism of PBO on fipronil toxicity

Preliminary experiments examining the toxicity of PBO to the susceptible and resistant larvae of *C. suppressalis* showed that the compound was toxic at doses greater than 30 µg/larva (data not shown). Synergism assays were therefore performed using fipronil in combination with PBO at either 15.0, 6.0, or 3.0 µg/larva. Table 2 shows that PBO at each concentration had a significant antagonistic effect on the toxicity of fipronil to susceptible larvae. Antagonism ratios (at the LD_50_) were 0.27–0.44-fold. However, PBO showed an obvious synergistic effect on the toxicity of fipronil to resistant larvae. Synergism ratios (at the LD_50_) were 1.85– 2.53-fold. At 6 µg/larva PBO had the strongest synergistic potential. Larval behavior after treatment of PBO alone were equivalent to the control, indicating an absence of toxic effects of PBO itself at the concentrations used for the synergism experiments.

**Table 1.**
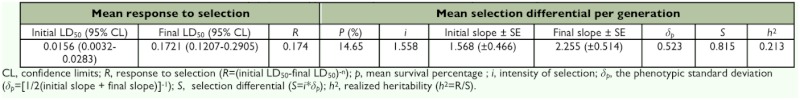
Estimation of resistance realized heritability (*h*^2^) of *C. suppressalis* to fipronil from laboratory-selection experiments.

**Table 2.**
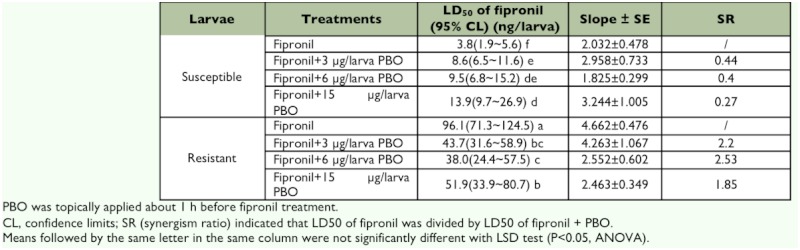
Synergism of PBO on toxicity of fipronil against fourth-instar S and R larvae of *C. supperssalis*.*

**Table 3.**
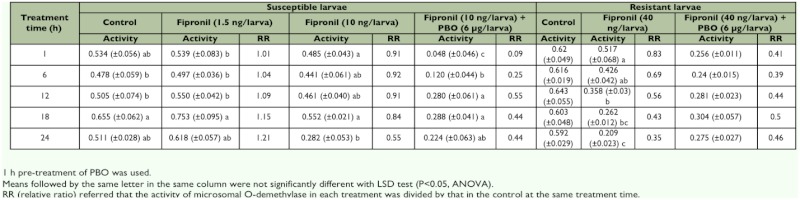
*Ex vivo* activity of microsomal *O*-demethylase from S and R larvae of *C. suppressalis* after treated by fipronil and fipronil+PBO.

### Time-effect synergism of PBO on fipronil toxicity

**Figure 1.**
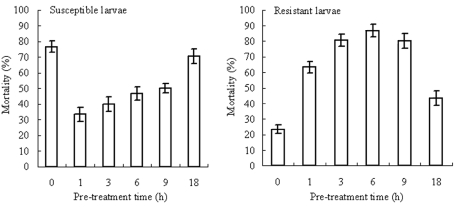
Effect of fipronil on the mortality of the susceptible and resistant larvae of *C. suppressalis* after pre-treatment with PBO (6 µg/larva). High quality figures are available online.

Assessment of the effects of PBO after 1, 3, 6, 9 and 18 h pre-treatment on the toxicity of fipronil on the fourth-instar susceptible and resistant larvae indicated that a dose of 6 µg/lalva was suitable for synergism experiments (Figure 1). PBO itself did not affect larval mortality at 24 and 48 h, but showed a differential effect on the toxicity of fipronil to the susceptible and resistant larvae. The presence of PBO decreased the effects of fipronil at10 ng/larva (at the LD_70_) on mortality of the susceptible larvae compared to fipronil alone, but with longer PBO pretreatment, the toxicity of fipronil to the susceptible larvae was gradually restored. However, the presence of PBO increased the toxicity of fipronil at a dose of 40 ng/larva (at the LD_20_) on the resistant larvae compared to fipronil alone. The application of PBO at 6 h pre-treatment exhibited the maximal synergism effect on the toxicity of fipronil to the resistant larvae.

### Activity of microsomal *O*-demethylase

The applications of fipronil and fipronil plus PBO significantly changed the activity of microsomal *O*-demethylase in the susceptible and resistant larvae. As shown in Table 3, fipronil at 1.5 ng/larva (at the LD_20_) slightly induced enzyme activity in susceptible larvae by 1.01–1.21-fold as compared with the control. After fipronil treatment at 10 ng/larva (at the LD_70_), enzyme activity in susceptible larvae gradually decreased as the incubation time was increased, and 6 µg/larva PBO caused a decrease in enzyme activity from 10.10- to 1.26-fold as compared to fipronil alone. Enzyme activity in the resistant larvae treated with fipronil at a dose of 40 ng/larva (at the LD_20_) decreased as the incubation time was increased, and was inhibited by 6 µg/larva PBO by 2.02- to 0.76-fold. These results show that effect of topical application of PBO on the activity of microsomal *O*-demethylase in the susceptible larvae was greater than in the resistant larvae.

## Discussion

Resistance risk assessment that reveals the maximum potential of a pest to develop resistance to the insecticide after selection has become an important way to avoid or at least postpone resistance problems in field (Lorinia et al. 2000; Keiding 1986). For example, a 300-fold increase of fipronil resistance in the population of diamondback moth and 430-fold fipronil-resistant housefly strain could be obtained by successive laboratory selection (Li et al. 2006; Michael et al. 2004). In this paper, the Wenzhou strain of *C. suppressalis* with 45.3-fold resistance to fipronil was obtained after 6-generations of discontinuous laboratory selection, the realized heritability (*h*^2^) showed that this strain possessed a moderately heritable phenotype and had potential to develop higher tolerance to fipronil. Thus, fipronil, an effective agent in IPM program for *C. suppressalis,* should be used more cautiously to avoid potential resistance development.

As is widely known, changes in metabolism and/or penetration of insecticides usually cause low levels of resistance in insects (Mohan et al. 2003; Kristensen et al. 2004; Goff et al. 2005; Li et al. 2006). The microsomal monooxygenase oxidative system is considered to be involved in the degradation of fipronil, as the formation of fipronil-sulfone, the predominant metabolite of fipronil, is dependent on this enzyme system (Brookhart et al. 1994; Scharf et al. 2000; Durham et al. 2002). A synergistic action would indicate the potential for using piperonyl butoxide to decrease the toxicity of fipronil that can be metabolised by P450 oxidative metabolism. It is interesting that the effect of piperonyl butoxide on the action of fipronil was synergistic in house flies (*Musca domestica*) (Scott et al. 1997), antagonistic in German cockroach (*Blattella germanica*) and European corn borer (*Ostrinia nubilalis*) (Durham et al. 2002; Valles et al. 1997), and had no effect in western corn rootworm (*Diabrotica virgifera virgifera*) (Scharfet al. 1999). In this paper, the application of PBO had a significant antagonistic effect on the toxicity of fipronil accompanied with decreasing the effects of fipronil on mortality of the susceptible larvae, whereas PBO had an obvious synergistic effect on the toxicity of fipronil to the resistant larvae causing a decreased LD_50_ value and an increased mortality compared to fipronil alone.

In the case of microsomal *O*-demethylase, the effect of PBO by topical application on enzyme activity in the susceptible larvae was greater than that in the resistant larvae. The different effects of PBO on the toxicity of fipronil and the activity of microsomal *O*-demethylase might have been caused by several possible mechanisms. Microsomal *O*-demethylase in the susceptible larvae that were obtained from Anhui strain and never exposed to fipronil, was sensitive to inhibition by PBO, so the lower conversion to fipronil-sulfone could lead to the decreased toxicity of fipronil in the susceptible larvae. Effects of PBO in the resistant larvae that were continuously selected by the LD_70_ dose of fipronil from the Wenzhou strain were non-metabolic, perhaps by effects on the penetration of fipronil. Pretreatment with PBO might facilitate the penetration of fipronil and cause higher amounts of internal accumulation of fipronil in the resistant larvae, therefore causing synergistic effects. Of course, these possibilities need to be examined.

Noppun et al. (1989) and Delorme et al. (1988) reported that reduced penetration was considered to be an important mechanism responsible for fenvalerate resistance in the diamondback moth (*Plutella xylostella*) and deltamethrin resistance in the beet armyworm (*Spodoptera exigua*). Fipronil resistance in the diamondback moth was inherited as an autosomal, incompletely recessive trait, and it was completely recessive at the highest dose of fipronil (Syyed et al. 2004). Therefore, high level resistance caused by target site insensitivity in an insect might abruptly occur during continuous use of firponil. Use of PBO could foster low levels of resistance caused by metabolism and/or penetration changes in *C. suppressalis* and might be useful during early application of fipronil in the field to extend the use of fipronil in integrated pest management strategies.

## Abbreviations

PBO: piperonyl butoxide;
MOD: microsomal *O*-demethylase;
PMSF: phenylmethyl sulfonyl fluoride;
NADPH: β-nicotinamide adenine dinucleotide 2′-phosphate reduced tetrasodium salt;
PTU: phenylthiourea;
EDTA: ethylenediamine tetraacetic acid;
DTT: 1,4-dithiothreitol;
*p*-NA: *p*-nitroanisole
